# Using *Bacillus amyloliquefaciens* for remediation of aquaculture water

**DOI:** 10.1186/2193-1801-2-119

**Published:** 2013-03-20

**Authors:** Fengxing Xie, Taicheng Zhu, Fengfeng Zhang, Ke Zhou, Yujie Zhao, Zhenghua Li

**Affiliations:** 1 Tianjin Institute of Industrial Biotechnology, Chinese Academy of Sciences, 32XiQiDao, Tianjin Airport Economic Park, Tianjin, 300308 People’s Republic of China; 2 Tianjin Research Center of Agricultural Biotechnology, Tianjin, 300192 People’s Republic of China; 3 Institute of Microbiology, Chinese Academy of Sciences, Beijing, 100101 People’s Republic of China

**Keywords:** Bacillus amyloliquefaciens, Aquaculture water remediation, Nitrogenous compound, Nitrite removal

## Abstract

Remediation of aquaculture water using microorganisms like *Bacillus* species is a burgeoning trend for the sustainable development of aquaculture industries. In this work, a *Bacillus amyloliquefaciens* strain (namely *B. amyloliquefaciens* HN), isolated from activated sludge of a polluted river, was evaluated for its potential in water remediation using simulated aquaculture water. *B. amyloliquefaciens* HN exhibited high tolerance towards 80 mg l^-1^ of nitrite-N and ammonia-N. It could effectively remove 20 mg l^-1^ of nitrite-N, but was inefficient in eliminating ammonia-N when the ammonia-N concentration was below 20 mg l^-1^. Further studies showed that the ammonia-N removal by *B. amyloliquefaciens* HN was more efficient at 30°C and 35°C than 25°C, and that maximum nitrite-N removal rate was achieved at pH 8.

## Introduction

The rapid expansion of intensive aquaculture industries, are often companied by rotted uneaten feed, sedimentation of feces and organic residues. The water quality rapidly deteriorates as a result. In particular, nitrogenous compounds such as ammonia and nitrite quickly build up, which are both harmful to fish and shrimp even at low concentrations (Crab et al. 2007; Mohapatra et al. 2012). Water exchange can be applied to maintain good water quality. However, frequent water exchange is not only laborious and costly, but also may incur disease causing agents and pollute nearby rivers and coastal areas (Mohapatra et al. 2012). Therefore, there is an urgent demand for cost-effective and environment-friendly approaches for remediation of aquaculture water.

In recent years, the use of microorganisms to improve water quality becomes a burgeoning trend (Ninawe and Selvin 2009; Verschuere et al. 2000). *Bacillus* species are widely used for water remediation because they are stable for long period due to spore formation, easily prepared by fermentation and possess antagonistic effects on pathogens (Hong et al. 2005). Strains belonging to several *Bacillus* species, such as *Bacillus subtilis*, *Bacillus cereus*, *Bacillus licheniformis*, *Bacillus pumilus* were isolated and evaluated for their potential as biological agents for water quality enhancement. Several strains with good nitrogen removal properties were thus found. To date, screening strains with good remediation characteristics still remains a fundamental step towards developing commercial microbial agents.

Previously, we isolated a *Bacillus amyloliquefaciens* strain, named as *B. amyloliquefaciens* HN from the activated sludge of a polluted river. This strain was shown to effectively remove nitrogenous compounds and grow in broad temperature, pH, and salts concentration in preliminary studies. Moreover, no previous studied has characterized the nitrogen removal ability of *B. amyloliquefaciens*. Therefore, the aim of this study is to evaluate *B. amyloliquefaciens* HN for its remediation properties using simulated aquaculture water.

## Materials and methods

### Strains and culture media

*B. amyloliquefaciens* HN was isolated from a polluted pool in Xiqing District, Tianjin. The strain was identified by Agricultural Culture Collection of China, and deposited at China General Microbiological Culture Collection Center (CGMCC No. 3261).

The culture medium (also referred as basal medium) for *B. amyloliquefaciens* HN contained (per liter): peptone 8 g, beef extract 3 g, soluble starch 5 g, NaCl 5 g (plus agar 15 g for solid medium). The simulated aquaculture water was prepared referring to Chen (2005) and LuzE. De-Bashanet (2002), which contained (per liter): beef extract 0.5 g, sucrose 0.5 g, NaCl 0.25 g, KH_2_PO_4_ 0.075 g.

### Nitrite and ammonia-N tolerance tests

Sodium nitrite and ammonium sulfate were added to the culture medium to make the final concentrations of nitrite-N 1, 5, 10, 20, 40, 80, and 100 mg l^-1^, respectively, and those of ammonia-N 10, 20, 40, 80, and 160 mg l^-1^, respectively. All the experiments were repeated thrice with the basal medium as control. Three milliliters of strain culture were inoculated into 250 ml shake flask with 100 ml culture media and cultured at 37°C.

### Removal tests of nitrite-N and ammonia-N

The simulated polluted water was prepared by making the final concentrations of nitrite-N 10, 20, 40, and 80 mg l^-1^, respectively, and the concentrations of ammonia-N 5, 10, 20, and 40 mg l^-1^, respectively. Three milliliters of strain culture were inoculated into 250 ml shake flask with 100 ml culture media and cultured at 37°C.

### Effects of different conditions on the removal of nitrite-N and ammonia-N

The model polluted water was prepared to make the initial concentration of nitrite-N was 10 mg l^-1^, and ammonia-N was 20 mg l^-1^. The inoculum volume was 3%, and cultured at different temperature (25°C, 30°C, and 35°C), and different initial pH (pH5.0, 6.0, 7.0, 8.0, and 9.0, adjusted by NaOH and HCl).

### Analysis methods

The Nesslar method was used for ammonia determination. The 1,2-ethanediamine, N-1-naphthalenyl-,dihydrochloride spectrophotometric method was used for the nitrite measurement (APHA, 2005). The nitrate concentration was measured using the salicylic (2-hydroxybenzoic) acid method (Cataldo et al. 1975). Cell concentration was determined by plate counting.

## Results

### Nitrite and ammonia-N tolerance tests for of *B. amyloliquefaciens* HN

**Figure 1:**
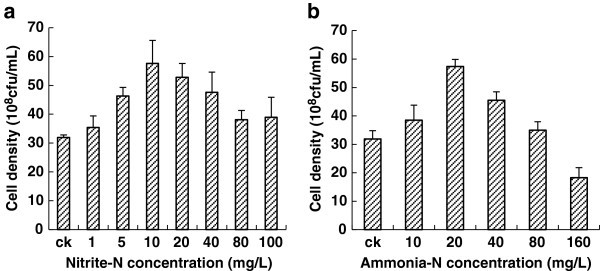
**Nitrite and ammonia-N tolerance tests for*B. amyloliquefaciens*HN. a**) Nitrite tolerance tests; **b**) Ammonia-N tolerance tests.

For Nitrite-N tolerance test (Figure 1a), cell concentrations of *B. amyloliquefaciens* HN with all tested nitrite-N concentrations were higher that of control, illustrating that the strain could tolerate wide range of nitrite-N. The results also showed that the presence of nitrite-N could promote the growth of *B. amyloliquefaciens*, especially at low concentrations (< 10 mg/L). The highest cell concentration was achieved with the nitrite-N concentration of 10 mg l^-1^, which is 80.1% higher than that of control.

For ammonia-N tolerance test (Figure 1b), all cell concentrations with tested ammonia-N concentrations were higher than that of control, except the one with 160 mg l^-1^ ammonia-N. The ammonia-N concentration of 20 mg l^-1^ resulted in the highest cell growth, 79.9% higher than that of control. The results indicated that *B. amyloliquefaciens* HN can tolerate ammonia-N ranging from 10 mg l^-1^ to 80 mg l^-1^. An ammonia-N concentration of 160 mg l^-1^ was shown to inhibit the growth of the strain.

### Nitrite-N and ammonia-N removal tests for *B. amyloliquefaciens* HN

**Figure 2:**
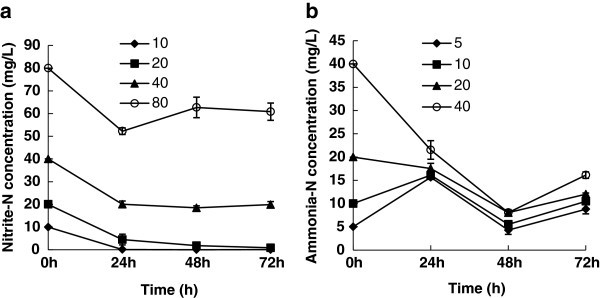
**Nitrite and ammonia-N removal tests for*B. amyloliquefaciens*HN. a**) Nitrite removal tests; **b**) Ammonia-N removal tests.

Figure 2a showed that the nitrite-N could not be detected within 24 h with an initial nitrite-N concentration of 10 mg l^-1^. 90% of the nitrite-N was removed in 24 h for 20 mg l^-1^, and almost 100% was removed in 48 h. 19.2 mg l^-1^ and 20.1 mg l^-1^ of nitrite-N (representing 53.8% and 21.6% of the total nitrite-N) was eliminated for an initial nitrite-N concentration of 40 mg l^-1^ and 80 mg l^-1^, respectively. The removal rate, however, did not significantly elevated with the time (the residue nitrite-N even increased a little for 80 mg l^-1^), suggesting that the maximum removal concentration of nitrite-N might be around 20 mg l^-1^.

With high initial ammonia-N concentrations (20 mg l^-1^ and 40 mg l^-1^) ,the removal rates of ammonia-N were significant, and maximum removal rates of 59.8% and 79.8% were reached at 48 h (Figure 2b). As for low initial ammonia-N concentrations (10 mg l^-1^ and 20 mg l^-1^), the removal efficiencies of ammonia-N were not efficient. During the time course, the residue ammonia-N levels fluctuated around 10 mg l^-1^, suggesting that *B. amyloliquefaciens* HN could not only utilize ammonia-N but also produce it.

### Effects of temperature on the nitrite-N and ammonia-N removal abilities of B. *amyloliquefaciens* HN

**Figure 3:**
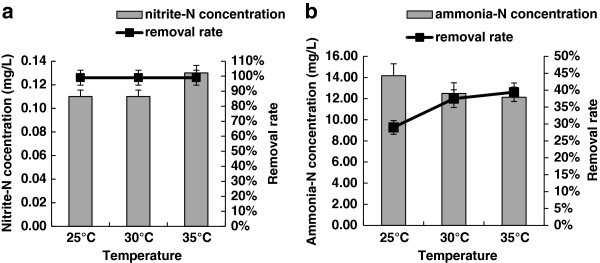
**Effects of different temperature on the removal rates of nitrite-N and ammonia-N by*B. amyloliquefaciens*HN.a**) Effects of temperature on nitrite-N removal rate; **b**) Effects of temperature on the ammonia-N removal rate.

The nitrite-N removal rates of *B. amyloliquefaciens* HN at 25°C, 30°C and 35°C all reached 99% (Figure 3a), suggesting temperature (over the experimental range) did not affect the removal of nitrite-N. The ammonia-N removal rates at 25°C, 30°C and 35°C were 29%, 37.5% and 39.4% respectively (Figure 3b), suggesting higher temperature might be preferable for effective removal of ammonia-N by *B. amyloliquefaciens* HN.

### Effects of pH on the nitrite-N and ammonia-N removal abilities of *B. amyloliquefaciens* HN

**Figure 4:**
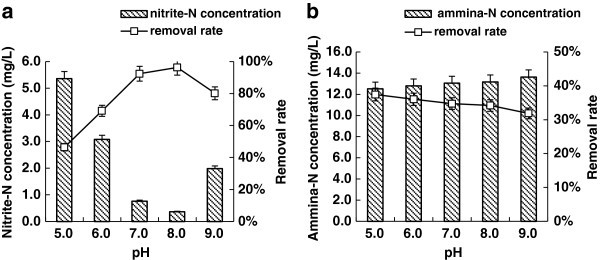
**Effects of pH on the removal rates of nitrite-N and ammonia-N. a**) Effects of pH on nitrite-N removal rate; **b**) Effects of pH on the ammonia-N removal rate.

Figure 4a showed that pH remarkably influenced the removal of nitrite-N by *B. amyloliquefaciens* HN. Nitrite-N removal rates increased significantly with increased pH value. A maximum removal rate of 96% could be reached at pH 8.0. Further increase of pH reduced the removal rate. Ammonia-N removal rates from pH 5.0 ~ 9.0 were between 32 ~ 38%, suggesting pH had no significant effects on the removal of ammonia-N (Figure 4b).

## Discussion

In this work, we investigated the potential of using *B. amyloliquefaciens* in the remediation of aquaculture water. Particularly, its remediation characteristics regarding nitrite and ammonia removal were evaluated. The accumulation of nitrite and ammonia is highly toxic to aquatic fauna. 0.1 ~ 10 mg l^-1^ of nitrite can cause 50% mortality (LC 50) of a number of fish and shrimp (Philips et al. 2002). Ammonia-N can be toxic to commercially cultured fish at concentrations above 1.5 mg l^-1^ (Crab et al. 2007).

*Bacillus* species are important candidates for developing commercial biological agents for nitrogen removal and water quality enhancement (Hong et al. 2005). Previously, a few studies reported that some strains of *B. subtilis* (Chen and Hu 2011; Meng et al. 2009), *B. lichenformis* (Meng et al. 2009) and *B. cereus* (Lalloo et al. 2007) exhibited strong nitrite removal ability. Physiological studies on *Bacillus* spp. also showed that *Bacillus* spp. could utilize nitrate and nitrite as alternative electron acceptors and nitrogen sources (Nakano et al. 1998; Hoffmann et al. 1998).

This study showed that an *B. amyloliquefaciens* strain, isolated from the activated sludge, was also a very efficient nitrite-N cleaner, which was able to completely remove 10 mg l^-1^ of nitrite-N present in the simulated aquaculture water within 24 h. In the past, *B. amyloliquefaciens* were applied in enzyme production (Wei et al. 2011), plant disease control (Alvindia and Natsuaki 2009; Arrebola et al. 2010) and food preservation (Wang et al. 2006). This is the first study on its potential application in improving water quality. It suggested that *B. amyloliquefaciens* might be an important alternative *Bacillus* species for nitrite removal.

The ammonia-N removal ability of *B. amyloliquefaciens* was not satisfying, because when the ammonia-N concentration was below 20 mg l^-1^, the removal rate was at best 39.4% and the ammonia-N concentration cannot be reduced below 10 mg l^-1^. Past literature seems to suggest that *Bacillus* species are not very efficient in ammonia removal, and no ammonia removal efficiency of a single *Bacillus* strain has been reported to exceed 90%. Therefore, to create a microbal agent which can simultaneously eliminate nitrite and ammonia, *B. amyloliquefaciens* NH can be formulated with efficient ammonia reducing bacteria, such as nitrifying bacteria (Meng et al. 2009).
